# Constitutively Nuclear FOXO3a Localization Predicts Poor Survival and Promotes Akt Phosphorylation in Breast Cancer

**DOI:** 10.1371/journal.pone.0012293

**Published:** 2010-08-20

**Authors:** Jie Chen, Ana R. Gomes, Lara J. Monteiro, San Yu Wong, Lai Han Wu, Ting-Ting Ng, Christina T. Karadedou, Julie Millour, Ying-Chi Ip, Yuen Nei Cheung, Andrew Sunters, Kelvin Y. K. Chan, Eric W.-F. Lam, Ui-Soon Khoo

**Affiliations:** 1 Department of Pathology, Li Ka Shing Faculty of Medicine, The University of Hong Kong, Hong Kong, China; 2 Cancer Research-UK Labs, Department of Surgery and Cancer, Imperial College London, Hammersmith Hospital Campus, London, United Kingdom; 3 Department of Veterinary Basic Sciences, Royal Veterinary College, London, United Kingdom; Baylor College of Medicine, United States of America

## Abstract

**Background:**

The PI3K-Akt signal pathway plays a key role in tumorigenesis and the development of drug-resistance. Cytotoxic chemotherapy resistance is linked to limited therapeutic options and poor prognosis.

**Methodology/Principal Findings:**

Examination of FOXO3a and phosphorylated-Akt (P-Akt) expression in breast cancer tissue microarrays showed nuclear FOXO3a was associated with lymph node positivity (p = 0.052), poor prognosis (p = 0.014), and P-Akt expression in invasive ductal carcinoma. Using tamoxifen and doxorubicin-sensitive and -resistant breast cancer cell lines as models, we found that doxorubicin- but not tamoxifen-resistance is associated with nuclear accumulation of FOXO3a, consistent with the finding that sustained nuclear FOXO3a is associated with poor prognosis. We also established that doxorubicin treatment induces proliferation arrest and FOXO3a nuclear relocation in sensitive breast cancer cells. Induction of FOXO3a activity in doxorubicin-sensitive MCF-7 cells was sufficient to promote Akt phosphorylation and arrest cell proliferation. Conversely, knockdown of endogenous FOXO3a expression reduced PI3K/Akt activity. Using MDA-MB-231 cells, in which FOXO3a activity can be induced by 4-hydroxytamoxifen, we showed that FOXO3a induction up-regulates PI3K-Akt activity and enhanced doxorubicin resistance. However FOXO3a induction has little effect on cell proliferation, indicating that FOXO3a or its downstream activity is deregulated in the cytotoxic drug resistant breast cancer cells. Thus, our results suggest that sustained FOXO3a activation can enhance hyperactivation of the PI3K/Akt pathway.

**Conclusions/Significance:**

Together these data suggest that lymph node metastasis and poor survival in invasive ductal breast carcinoma are linked to an uncoupling of the Akt-FOXO3a signaling axis. In these breast cancers activated Akt fails to inactivate and re-localize FOXO3a to the cytoplasm, and nuclear-targeted FOXO3a does not induce cell death or cell cycle arrest. As such, sustained nuclear FOXO3a expression in breast cancer may culminate in cancer progression and the development of an aggressive phenotype similar to that observed in cytotoxic chemotherapy resistant breast cancer cell models.

## Introduction

Breast cancer is the most common malignancy in women and represents one of the major causes of death worldwide. Endocrine agents have become the primary adjuvant treatment for breast cancer [1], [2]. Tamoxifen, a selective estrogen receptor modulator (SERM), is widely used for treating estrogen receptor α (ERα) positive breast cancer patients. Although most ERα positive breast cancer patients initially respond to tamoxifen therapy, approximately half of the patients will eventually develop resistance and relapse, following long-term treatment [1], [3], [4], [5]. The effects of Tamoxifen in breast tissues result from its ability to bind to the ligand-binding domain of the ERα, thereby antagonizing the proliferative potential of estrogen [1], [2]. Other similar strategies, including estrogen withdrawal and pure estrogen antagonism, have also been employed to block the mitogenic effects of estrogen on breast cancer cells. However, most tumour cells will eventually adopt as yet unclear mechanisms to develop insensitivity or resistance [6], [7]. Although the predictive markers for endocrine therapy response, namely expression of ERα and progesterone receptor are popularly used for determining clinical management [8], [9], survival signaling pathways regulated by PI3K, Akt (also called PKB) and PTEN are also found to be crucial in drug resistance [4], [10], [11], [12], [13], indicating that multiple biomarkers are required to fully predict the development of drug resistance.

For those breast cancer patients who relapse after endocrine treatment or those with tumours that do not express hormone receptors, chemotherapeutic agents, including taxenes (eg. Paclitaxel and Docetaxel) and anthracyclins (eg. Epirubicin and Doxorubicin), represent important backup treatment options [1], [2]. These systemic chemotherapy backup treatments are also essential for patients with metastatic or advanced stage breast cancer. However, for those breast cancer patients who are unresponsive or have subsequently become resistant to taxane and anthracycline-based chemotherapies, their treatment options are limited and their outlook is poor. Although taxane and anthracycline-based chemotherapeutic regimens can stop cancer cells from multiplying, their non-specific actions damage normal healthy cells, curtailing further treatment. It is therefore important to develop good predictors for chemotherapy response, so that these agents are only used for treating patients responsive to the treatment; whereas non-responsive patients can be switched to alternative treatments at an early stage.

Previous studies have shown that increased Akt activity can promote breast cancer cell survival and therapeutic resistance [6], [7], [10], [11]. Furthermore, the PI3K-Akt signaling pathway has also been demonstrated to play a crucial role in the development of tamoxifen resistance [10], [11], [12]. FOXO transcription factors (FOXO3a, FOXO1 and FOXO4) are downstream targets of the PI3K-Akt pathway, which play a vital role in a variety of cellular processes, such as cellular differentiation, tumour suppression, metabolism, cell cycle arrest, cell death and protection from stress [14]. Akt phosphorylates three sites on FOXO proteins leading to their nuclear exclusion and inactivation, which is known to associate with tumorigenesis and cancer progression [15].

Most studies on FOXO3a in breast cancer have been conducted on breast cancer cell lines or animal models. For the few expression studies of FOXO3a in breast cancer patient samples, the results have been conflicting. Hu *et al* (2004) [16] have found cytoplasmic expression of FOXO3a correlated with phosphorylated Akt (P-Akt) and associated with poor survival in breast cancer. Yet, another study found that nuclear rather than cytoplasmic FOXO3a was significantly associated with lymph node metastasis [17]. To explore the potential role of the Akt-FOXO3a axis in breast cancer prognosis, we studied the expression of FOXO3a and P-Akt in a tissue microarray of 130 breast cancer cases. Contrary to previous findings of Hu *et al* (2004) [16], we found predominant nuclear accumulation of FOXO3a was associated with poor prognosis and active P-Akt expression, suggesting an uncoupling of the Akt-FOXO3a axis during breast cancer progression and development of drug resistance. To validate these findings, we studied FOXO3a nuclear expression and its consequence in a panel of chemotherapy sensitive and resistant breast cancer cell lines.

## Materials and Methods

### Tissue Microarray

One hundred and thirty-three cases of breast cancer diagnosed between the years 1992 to 2001 with clinical follow up data were retrieved from the records of the Department of Pathology, Queen Mary Hospital of Hong Kong. The patients' ages at diagnosis ranged from 30 to 90 years old, with a mean of 53 years. Histological sections of all cases were reviewed by the pathologist, the representative paraffin tumour blocks chosen as donor block for each case and the selected areas marked for construction of tissue microarray blocks. Of all cases, 38 had non-tumour tissues available for comparison with their tumour counterparts in protein expression studies. A total of 120 could be assessed and scored for FOXO3a and P-Akt expression. The expression pattern and subcellular localization were correlated with histological type, histological grade, clinical stage, estrogen and progestrogen receptor status, HER2 oncoprotein overexpression, lymph node metastasis and survival time.

### Immnohistochemistry

Tissue sections were deparaffinized and rehydrated by incubation with xylene and decreasing concentrations of ethanol. Citrate buffer (0.01 M, pH 6.0) was used for antigen retrieval. The slides were immersed into 3% H_2_O_2_/methanol for 10 min at room temperature to quench endogenous peroxidase. After rinsing in 0.05% Tween in PBS (PBST) twice, a previously described primary polyclonal FOXO3a specific antibody [18], [19] diluted at 1∶1400 and P-Akt (Thr308) polyclonal (Cell Signaling, New England Biolabs, Hitchin, UK) diluted at 1∶100 were separately added to each section and incubated at 4°C overnight. The slides were then washed in PBST and 4–5 drops of DAKO Polymer were applied on each section and incubated at room temperature for 30 min. After washing, Chromogen DAB/substrate reagent was added onto the slides and the slides incubated for a further 3 min. Finally, the slides were dehydrated with increasing concentrations of ethanol followed by clearing in xylene and then mounted.

The staining of FOXO3a and P-Akt were assessed by light microscope visualized at high power (X170). The staining intensity and percentage of staining in the cytoplasm and the nucleus were scored in a semi-quantitative fashion. The intensity of staining was scored as follows: 1 =  weak, 2 =  moderate, 3 =  strong. The percentage of cells positively stained was scored as follows: 1≤25%, 2≤50%, 3≤75%, 4>75%. For each case, a final score from the nucleus and the cytoplasm was obtained by multiplying the score of intensity with the score of percentage, 12 being the maximum final score. To avoid subjectivity in evaluation, scoring was done by two independent individuals.

### Statistical Analysis

Statistical analysis was performed using the SPSS programme version 16. Correlation of FOXO3a or P-Akt expression to clinical and histological data was assessed by Chi-square test or Fisher's- exact test where applicable. Survival analysis was assessed by Kaplan Meier analysis using log-rank test. P-values of less than or equal to 0.05 were considered statistically significant.

### Cell Lines and Cell Culture

The human breast cancer cell lines MCF-7, BT474 and MDA-MB-231 originated from the American Type Culture Collection and were acquired from the Cell Culture Service, Cancer Research UK (London, UK), where they were tested and authenticated. All cells were cultured in DMEM, while MDA-MB-231-FOXO3a(A3):ER was cultured in DMEM-phenol red free, all supplemented with 10% foetal bovine serum, 2 mmol/L glutamine and 100 units/mL antibiotics (penicillin and streptomycin) (Sigma, Poole, UK), in a humidified incubator at 37°C with 5% CO_2_. The MCF-7DOX^R^ cell line is a doxorubicin resistant cell line derived from parental MCF-7 cells. Two estrogen-independent and tamoxifen-resistant cell lines, LCC2 and LCC9 were derived from MCF-7/LCC1 by stepwise *in vitro* selection of prolonged tamoxifen (LCC2) and fulvestrant (LCC9) treatment as described [20], while R27 is also a tamoxifen resistant cell line derived from parental MCF-7 (LCC1). Following *in vitro* selection, they were maintained in modified IMEM-phenol red free with 5% charcoal-stripped FBS. All the tamoxifen resistant cell lines were kindly provided by Dr Robert Clarke (Georgetown University Medical School, Washington. D.C.).

### Sulforhodamine B (SRB) assay

Approximately 3000 cells were seeded in each well of the 96 well plates. After culture, 100 µl of trichloroacetic acid was added to each well and incubated for 1 h at 4°C. The plates were then washed with deionised water for three times, before incubation at RT for 1 h with 0.4% SRB in 1% acetic acid. The plates were then washed with deionised water and air-dried. 10 mM Tris base was then added to the wells to solubilise the bound SRB dye, and the plates were then read at 492 nm using the Anthos 2001 plate read (Jencons Scientific Ltd, Leighton Buzzard, UK).

### Cell Cycle Analysis

Cell cycle analysis was done by propidium iodide (PI) staining Cells were seeded and serum starved for 24 h. With or without tamoxifen treated cells were trypsinized and then fixed in cold 70% ethanol after PBS wash. All the fixed cells were incubated in PBS with 5 µg/mL propidium iodide, 0.1 mg/mL RNase A and 0.5% Triton X-100 for 45 min at 37°C before analysis using a Becton Dickinson FACS analyzer (Oxford, UK). The cell cycle data were further analyzed by WinMDI 2.9.

### Drug Treatment Assay

For drug treatment, all cell lines were cultured in DMEM with 10% FBS, 2 mmol/L glutamine and 100 units/mL antibiotics (penicillin/streptomycin). Exponentially growing cells were incubated with 0.1 µM tamoxifen or 1 µM doxorubicin for the indicated time course, unless specified otherwise.

### Western Blot Analysis and Antibodies

Cells were lysed and SDS-PAGE gel electrophoresis was performed as previously described [21]. Harvested cells were lysed in protein lysis buffer (1% NP-40, 150 mM NaCl, 50 mM, 50 mM Tris-HCl (pH 7.4), 0.5% Sodium deoxycholate, 0.1% SDS, 2 mM EDTA, 1 mM PMSF and protease inhibitors (“Complete” protease inhibitor mixture, as instructed by the manufacturer Roche Applied Science, Lewes, UK) and the protein concentration was determined by Bio-Rad Dc protein assay (Bio-Rad, Hemel Hempstead, UK). Twenty micrograms of each protein sample were separated by SDS-PAGE, electrophoretically transferred onto Protran nitrocellulose membranes (Schliecher and Schuell, Dassel, Germany). After blocking in 5% BSA/TBST for 1 h at room temperature, membranes were probed with the respective primary antibodies at 4°C overnight and then detected using horseradish peroxidase linked anti-mouse, anti-goat or anti-rabbit conjugates as appropriate (DAKO, Ely, UK.), and visualized using the ECL detection system (Amersham Biosciences, Amersham, UK). Primary antibodies used were P-FOXO3a (Thr32), FOXO3a, P-Akt (Ser473) and Akt (all from Cell Signaling, UK), Lamin B (C-20) and β-tubulin (H-235) (Santa Cruz Biotechnology,Wiltshire, UK). The expression of FOXO isoforms has previously been studied in these breast cancer cell lines, and FOXO3a has been shown to be the predominant species [22].

### Nuclear and cytoplasmic lysate extraction

The fractionation was done by using NE-PER Nuclear and Cytoplasmic Extraction Reagents (Thermo Fisher, Horsham, UK) following the manufacturer's protocol.

### Transfection of FOXO3a expression vector

The FOXO3a expression vector pECE-TM-FOXO3a (FOXO3a (A3)), triple mutant FOXO3a in which all three P-Akt phosphorylation sites were mutated to alanine), encoding constitutively active FOXO3a, was obtained from Addgene (Cambridge, MA, USA). The FOXO3a(A3) insert was then cloned into vector pcDNA3.1(+) (Invitrogen, Paisley UK) for further study. For overexpression assay, MCF-7 cells were seeded into 24-well 24 h before transfection. Two microliter lipofectamine 2000 (Invitrogen, UK) and 0.8 µg FOXO3a (A3), plasmid were used for each well.

### Gene Silencing with Small hairpin RNAs (shRNAs)

For gene silencing, cells were seeded in 24-well plates and transfected with 0.8 µg FOXO3a shRNA (SABiosciences, USA) using lipofectamine 2000 system (Invitrogen). After 24 hours, total RNA was extracted to check knockdown efficiency by real-time quantitative PCR while total protein was used in Western blot assay.

### Real-time Quantitative PCR (RTQ-PCR)

TRizol reagent (Invitrogen) was used for total RNA extraction. One microgram of total RNA was reverse-transcribed into cDNA using High Capacity RNA-to-cDNA Master Mix (Applied Biosystems, Brackley, UK). The cDNA was then used as the template in the real-time quantitative PCR analysis while gene TBP was used as the endogenous control. The gene-specific primer pairs were showed in Table 1. Detection of FOXO3a and PIK3CA transcription level was performed with Power SYBR Green kit (Applied Biosystems, UK) with ABI 7800 system (Applied Biosystems, UK).

**Table 1:** Gene-specific primer pairs.

Primer	Sequence
FOXO3a-forward	5′-TCTACGAGTGGATGGTGCGTT-3′
FOXO3a-reverse	5′-CGACTATGCAGTGACAGGTTGTG-3′
PIK3CA-forward	5′-AAATGAAAGCTCACTCTGGATTCC-3′
PIK3CA-reverse	5′-TGTGCAATTCCTATGCAATC-3′
TBP-forward	5′-ACGAACCACGGCACTGAT-3′
TBP-reverse	5′-AACCCAACTTCTGTACAACTCTAGCA-3′

### Immunofluorescent Staining

Cells were subjected to anti-FOXO3a staining. Briefly, cells were fixed with 4% paraformaldehyde (Sigma, UK), permeabilized with 0.1%Triton X-100 in 10% FCS for 10 min. Samples were then blocked with 5% goat serum for 30 min and then incubated overnight with the primary rabbit anti-FOXO3a (Cell Signaling, UK). Following washes with PBS, secondary goat anti-rabbit IgG-FITC (1∶500, Invitrogen) was added to the samples for an hour. Cells were counterstained with 4′, 6′-diamidino-2-phenylindole (DAPI; Sigma UK) before mounting. Images were captured and quantified using the Zeiss Axiovert 100 confocal laser scanning microscope and software Zeiss LSM 500 (Zeiss Ltd).

## Results

### Nuclear FOXO3a expression in breast cancer tissue is significantly associated with lymph node metastasis and poor survival in invasive ductal carcinoma

**Figure 1:**
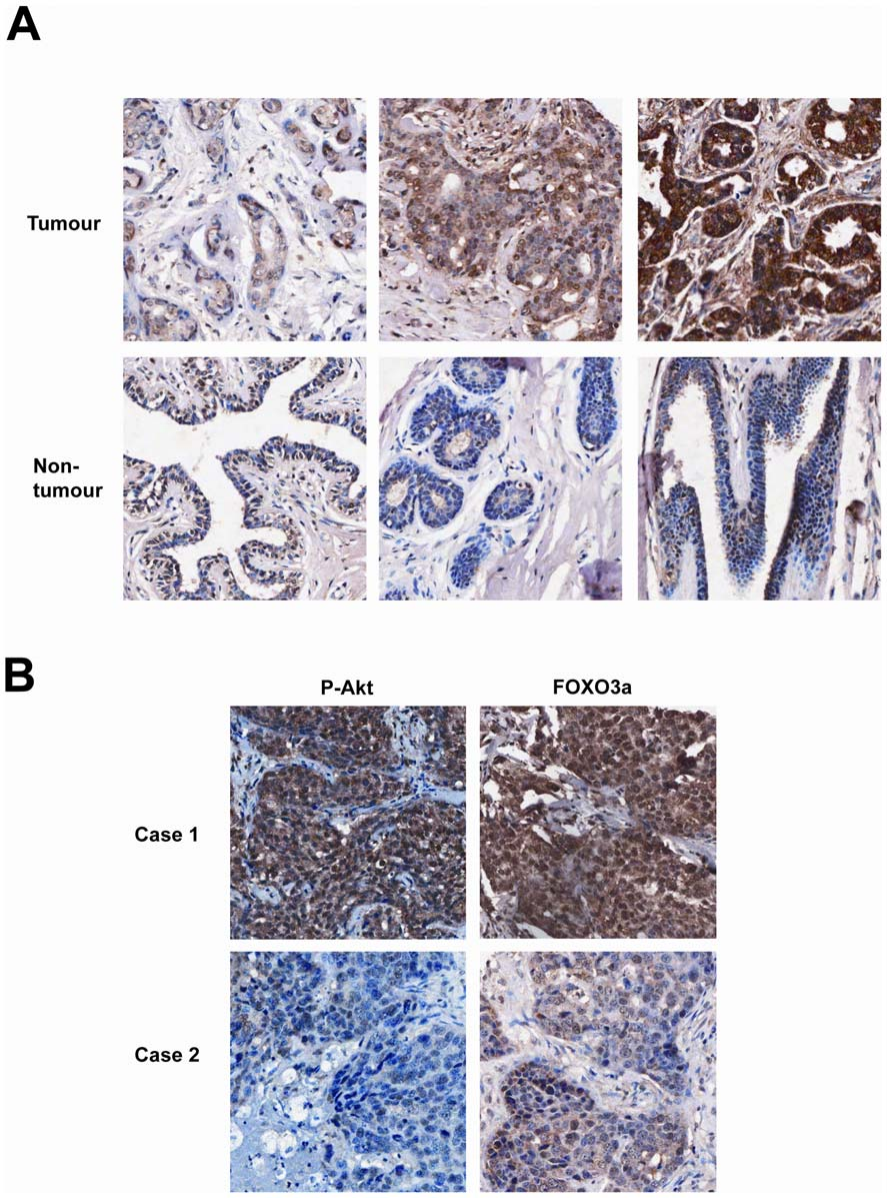
Representative expression patterns of FOXO3a and P-Akt in tissue microarray. Tumour tissue samples obtained from breast cancer patients that had been formalin-fixed and paraffin-embedded were immunohistochemically stained with FOXO3a and P-Akt (Thr308) antibodies using the streptavidin-biotin-peroxidase technique. A) Representative FOXO3a staining patterns in both tumour and non-tumour cases (magnification ×170). B) Two representative tumour cases showing corresponding FOXO3a and P-Akt staining patterns (magnification ×170). Case 1 shows high cytoplasmic pAkt staining and strong cytoplasmic and nucleus FOXO3a staining. Case 2 shows weak pAkt and FOXO3a staining.

To explore the potential role of the Akt-FOXO3a signaling axis as prognostic markers in breast cancer, the expression patterns of FOXO3a and P-Akt were studied using immunohistochemical staining on a panel of breast cancer samples (Table 2). The staining of FOXO3a revealed both nuclear and cytoplasmic localization in tumours (Table 3). Representative expression patterns in both tumour and non-tumour samples were shown in Figure 1A. Stronger cytoplasmic FOXO3a staining was observed to be common in tumour samples (P<0.0001, Chi-Square test). FOXO3a cytoplasmic staining (p<0.0001, Chi-Square test) was also significantly associated with P-Akt staining, which is supportive that the activated P-Akt can negatively regulate FOXO3a and relocate it from the nuclear to cytoplasm in most breast cancer samples.

**Table 2:** Histological types of tumour samples used in tissue microarray.

Pure ductal cell carcinoma in situ (pure DCIS)	10
Invasive ductal carcinoma	94
Invasive lobular carcinoma	2
Pure tubular or cribriform	1
Tubular mixed	5
Pure mucinous carcinoma	2
Medullary or atypical medullary	1
Others*	5
Total	120

*Others: 3 cases of mixed lobular and ductal carcinoma, 1 case of intraduct papillary carcinoma and 1 case of carcinosarcoma.

**Table 3:** FOXO3a immunoreactivity in all cases.

Tumour cases			
Nuclear staining	No. of cases	Cytoplasmic staining	No. of cases
negative	85	negative	4
N score ≤6	25	C score ≤6	24
N score >6	10	C score >6	92
Total	120	Total	120

Non-tumour cases			
Nuclear staining	No. of cases	Cytoplasmic staining	No. of cases
negative	0	negative	17
N score ≤6	27	C score ≤6	14
N score >6	11	C score >6	7
Total	38	Total	38

**Figure 2:**
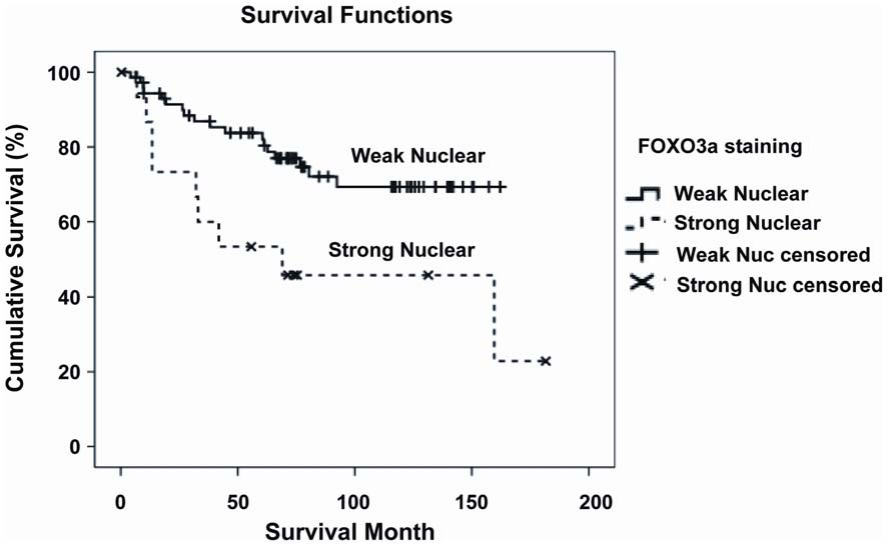
Survival Analysis with nuclear FOXO3a staining in invasive ductal carcinoma cases. Of the 120 cancer samples assessed, there were 94 cases of invasive ductal carcinoma. The correlation between nuclear FOXO3a expression and survival was studied using Kaplan Meier analysis (P = 0.014) and was considered significant at p<0.05.

The majority of tumour samples are invasive ductal carcinoma. Therefore, we re-analyzed the above parameters for cases of invasive ductal carcinomas only, and found that the nuclear staining of FOXO3a was correlated with lymph node positivity (p = 0.052, Chi-Square test). Furthermore, FOXO3a nuclear staining was also significantly associated with shorter survival time (p = 0.014, log-rank test) (Fig. 2). Interestingly, using the comparison scoring system N> = C or C>N as previously described [16], the N> = C FOXO3a staining of all cases irrespective of histological types was positively associated with the P-Akt staining (p = 0.039, Chi-square test), suggesting uncoupling of the Akt-FOXO3a signaling axis is associated with lymph node metastasis and poor survival in invasive ductal carcinoma. Representative stainings are showed in Figure 1B. Together these findings indicate that the function of FOXO3a and its control by Akt have been deregulated in breast cancer associated with lymph node metastasis and poor survival, indicative of cancer progression and treatment failure, respectively.

### Tamoxifen-resistant breast cancer cell lines are sensitive to doxorubicin treatment

**Figure 3:**
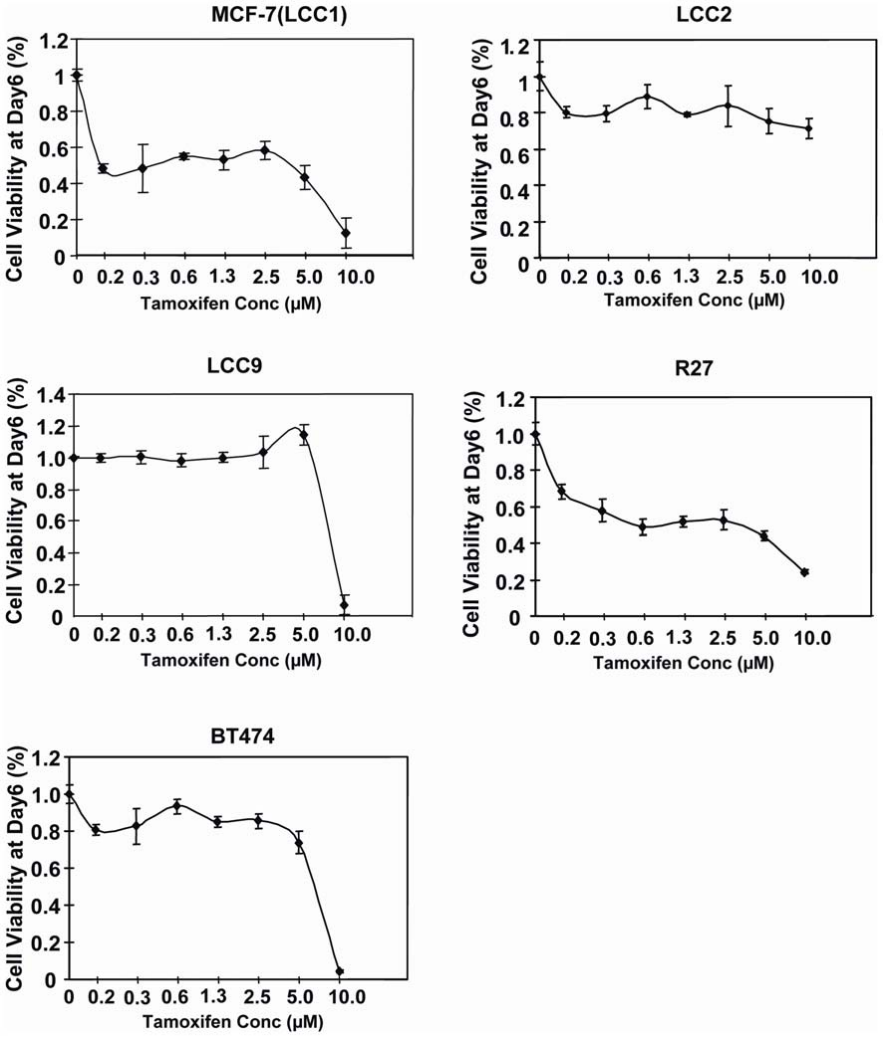
Effects of tamoxifen on cell proliferation of a panel of breast carcinoma cell lines. MCF-7(LCC1), LCC2, LCC9, R27 and BT474 cells were treated with 0 to 10 µM of tamoxifen for 6 days. Cell proliferation was determined by SRB assay. Points, mean of three independent experiments; bars, SD.

**Figure 4:**
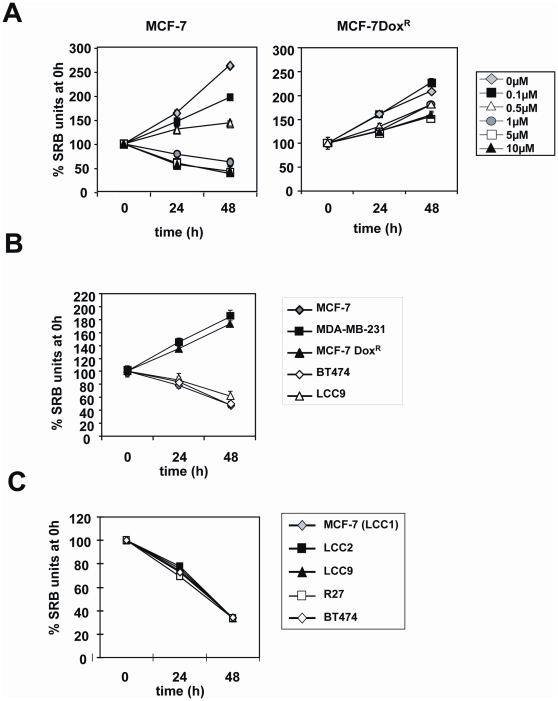
Effects of doxorubicin on cell proliferation of a panel of breast carcinoma cell lines. A) MCF-7 and the derived MCF-7 Dox^R^ cells were treated with 0 to 10 µM of doxorubicin for 0, 24, and 48 h. B) MCF-7, MCF-7 Dox^R^, MDA-MB-231, BT474, and LCC9 cells were treated with 1 µM of doxorubicin for 0, 24, and 48 h. C) MCF-7(LCC1), LCC2, LCC9, R27 and BT474 cells were treated with 1 µM of doxorubicin for 0, 24, and 48 h. Cell proliferation was determined by SRB assay. Points, mean of three independent experiments; bars, SD.

In order to confirm and explore further the immunohistochemical findings from patient samples, we sought to validate our findings in breast cancer tissue culture cell models. To this end, we first studied the drug resistance of a panel of chemotherapy (i.e. tamoxifen and doxorubicin) sensitive and resistant breast cancer cell lines. Proliferative assay results indicated that while the parental MCF-7 cell line is sensitive to tamoxifen, other MCF-7 derivative clones, LCC2, LCC9, R27 as well as the BT474 cells are resistant to tamoxifen treatment from concentrations 2.5 to 5 µM (Fig. 3). We also generated a cytotoxic chemotherapy resistant MCF-7 cell line through continuous exposure up to 10 µM doxorubicin, and proliferative analysis confirmed that the proliferation of this MCF-7Dox^R^ cell pool is not significantly affected by doxorubicin treatment (Fig. 4A). In contrast, the proliferation of the parental MCF-7 cells was severely inhibited when incubated with > = 1 µM of doxorubicin. Similarly, the multidrug resistant breast carcinoma cell line MDA-MB-231 was also resistant to 1 µM of doxorubicin, a dose that effectively inhibits the parental MCF-7 cell proliferation (Fig. 4B). Notably, the tamoxifen resistant BT474 cells as well as the MCF-7 derived lines, including LCC2, LCC9 and R27, were all found to be sensitive to doxorubicin treatment at 10 µM (Fig. 4C). The MCF-7 Dox^R^ and MDA-MB-231 may represent breast cancers that have progressed and become drug resistant. This finding corroborates with the clinical evidence that breast cancers which failed endocrine treatments are often intrinsically sensitive to cytotoxic chemotherapy.

### Predominant nuclear FOXO3a localization in doxorubicin- but not tamoxifen-resistant breast cancer cell lines

**Figure 5:**
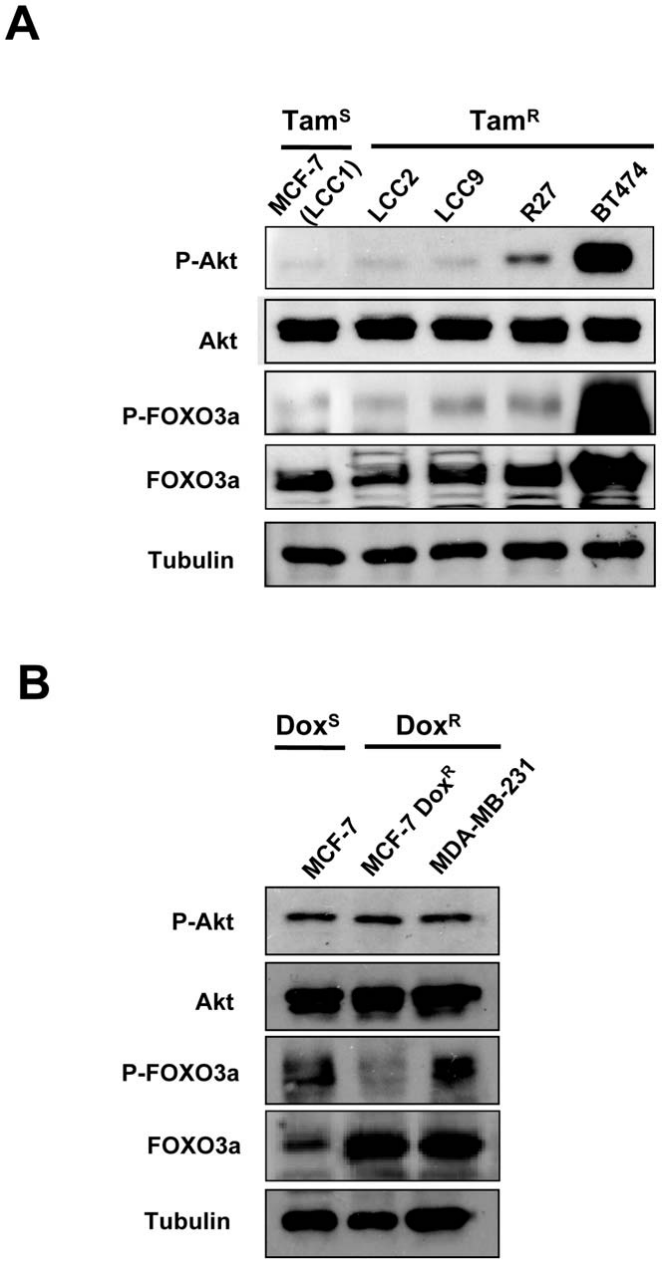
Expression of total and phosphorylated Akt and FOXO3a in a panel of tamoxifen and/or doxorubicin sensitive and resistant breast carcinoma cell lines. The tamoxifen sensitive MCF-7(LCC1), and resistant LCC2, LCC9, R27 and BT474 cells as well as the doxorubicin sensitive MCF-7 and resistant MCF-7-Dox^R^ and MDA-MB-231 cells were cultured in normal growth medium and used for Western blot analysis for P-Akt (Ser473), total Akt, P-FOXO3a (Thr32), total FOXO3a and β-tubulin.

To explore the potential role of FOXO3a and its upstream regulator Akt in the development of drug resistance, we first examined the basal expression levels of both the total and phosphorylated forms of FOXO3a and Akt in the drug sensitive as well as resistant breast cancer cell lines. Western blot analysis showed that the tamoxifen sensitive MCF-7 (LCC1) and resistant cell lines LCC2, LCC9 and R27 expressed comparable levels of P-Akt (Ser-473), FOXO3a and P-FOXO3a (Thr-32) (Fig. 5A), suggesting elevated P13K-Akt activity in both the endocrine sensitive and resistant cells. In contrast, BT474 cells expressed higher levels of P-Akt (Ser-473), and P-FOXO3a (Thr-32) but also more FOXO3a. Interestingly, P-Akt (Ser-473) and total Akt were expressed at similar levels in the doxorubicin sensitive MCF-7 and resistant MCF-7Dox^R^ and MDA-MB-231 cells (Fig. 5B). By comparison, the expression levels of P-FOXO3a (Thr-32) were low in the MCF-7Dox^R^ while the expression levels of total FOXO3a were high in both the MCF-7Dox^R^ and MDA-MB-231 cells. These results indicate that substantial amounts of FOXO3a were not phosphorylated and the Akt-FOXO3a axis is uncoupled in the doxorubicin resistant cells.

**Figure 6:**
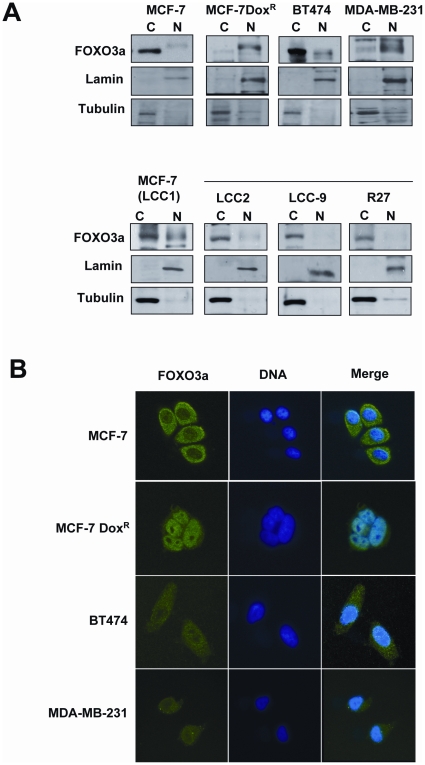
Nuclear location of FOXO3a in a panel of tamoxifen and/or doxorubicin sensitive and resistant breast carcinoma cell lines. The tamoxifen sensitive MCF-7(LCC1), and resistant LCC2, LCC9, R27 and BT474 cells as well as the doxorubicin sensitive MCF-7 and resistant MCF-7-DoxR and MDA-MB-231 cells were cultured in normal growth medium and used for studies of the subcellular localization of FOXO3a. A) Cytoplasmic and nuclear protein lysates were prepared and protein expression levels were analyzed by Western blotting using antibodies against specific antibodies against FOXO3a, lamin B1 and and β-tubulin, B) The cells were cultured on sterile coverslips, before being fixed in 4% formaldehyde. FOXO3a expression was visualized with a specific rabbit polyclonal antibody against FOXO3a followed by the addition of ALEX488 (green) labeled anti-rabbit antisera. DAPI (blue) were also applied to visualize the nuclei.

We next investigated the subcellular distribution of FOXO3a in the cytoplasmic and nuclear extracts from these drug sensitive and resistant breast cancer cell lines (Fig. 6A). Lamin B and tubulin were also probed as controls for effective nuclear and cytoplasmic fractionation respectively. While FOXO3a was detected at considerably higher levels in the cytosolic fractions compared with the nuclear fractions in all doxorubicin sensitive breast cancer, FOXO3a expression was found at appreciably higher levels in the nuclear fractions in the doxorubicin resistant breast cancer cells MCF-7Dox^R^ and MDA-MB-231. These results suggest that FOXO3a expression was primarily nuclear in doxorubicin-resistant cells, but was predominantly cytoplasmic in doxorubicin-sensitive cells. This result was also observed in tamoxifen-resistant BT474, LCC2 and LCC9 cell lines (Fig. 6A).

To confirm the subcellular fractionation results, immunofluorescent staining was then used to directly visualize the subcellular distribution of FOXO3a in the doxorubicin sensitive MCF-7 and BT474 and the drug resistant MCF-7Dox^R^ and MDA-MB-231 cells (Fig. 6B). The results showed that FOXO3a staining was primarily located in the cytoplasm of the doxorubucin sensitive cell lines, MCF-7 and BT474. In contrast, FOXO3a was expressed predominantly in the nucleus in doxorubucin resistant MCF-7Dox^R^ and MDA-MB-231 cells, further suggesting that the development of cytotoxic drug resistance may be associated with nuclear FOXO3a localization. These findings are consistent with the immunohistochemical staining results that increased Akt phosphorylation coupled with high levels nuclear FOXO3a expression is associated with poor prognosis in breast cancer.

### Doxorubicin induces FOXO3a nuclear relocation in drug sensitive cells

**Figure 7:**
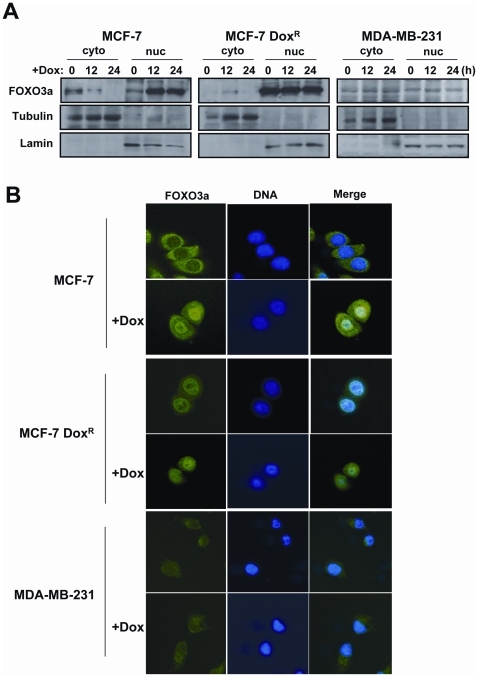
Doxorubicin treatment causes a nuclear relocation of FOXO3a expression. The doxorubicin sensitive MCF-7 and resistant MCF-7-Dox^R^ and MDA-MB-231 cells were treated with 1 µM doxorubicin. A) Cytoplasmic and nuclear protein lysates were prepared at the times indicated after doxorubicin treatment and protein expression levels were analyzed by Western blotting using antibodies against specific antibodies against FOXO3a, lamin B1 and and β-tubulin, B) The cells were cultured on sterile coverslips and treated for 16 h with or without 1 µM doxorubicin, before being fixed in 4% formaldehyde. FOXO3a expression was visualized with a specific rabbit polyclonal antibody against FOXO3a followed by the addition of ALEX488 (green) labeled anti-rabbit antisera. DAPI (blue) were also applied to visualize the nuclei.

To explore the potential role of FOXO3a in drug response and resistance, FOXO3a expression was examined in the cytoplasmic and nuclear fractions of the sensitive MCF-7 and the resistant MCF-7Dox^R^ and MDA-MB-231 cells in response to doxorubicin treatment. The Western blot results showed that FOXO3a expression increased in the nucleus, with a reciprocal decrease in levels in the cytoplasm in the sensitive cells, 12 and 24 h following doxorubicin treatment (Fig. 7A). Contrarily, FOXO3a expression levels remained unchanged in both the cytoplasmic and nuclear fractions of the resistant MCF-7DoxR and MDA-MB-231 cells in response to doxorubicin. To confirm the subcellular fractionation results, immunofluorescent staining was again used to gauge the subcellular distribution of FOXO3a in the doxorubicin sensitive MCF-7 and the drug resistant MCF-7DoxR and MDA-MB-231 cells in response to doxorubicin treatment (Fig. 7B). The results showed that FOXO3a relocated to the nucleus in the MCF-7 cells following 16 h of doxorubicin treatment, while the subcellular expression patterns of FOXO3a remained largely unchanged in the doxorubicin resistant cells. Together these results suggested that doxorubicin causes nuclear relocation of FOXO3a in the drug sensitive breast cancer cells, and confirmed our previous data that FOXO3a expression is predominantly nuclear in the resistant cells.

### Ectopic FOXO3a expression results in proliferation arrest and enhances Akt phosphorylation in cytotoxic drug sensitive cells

**Figure 8:**
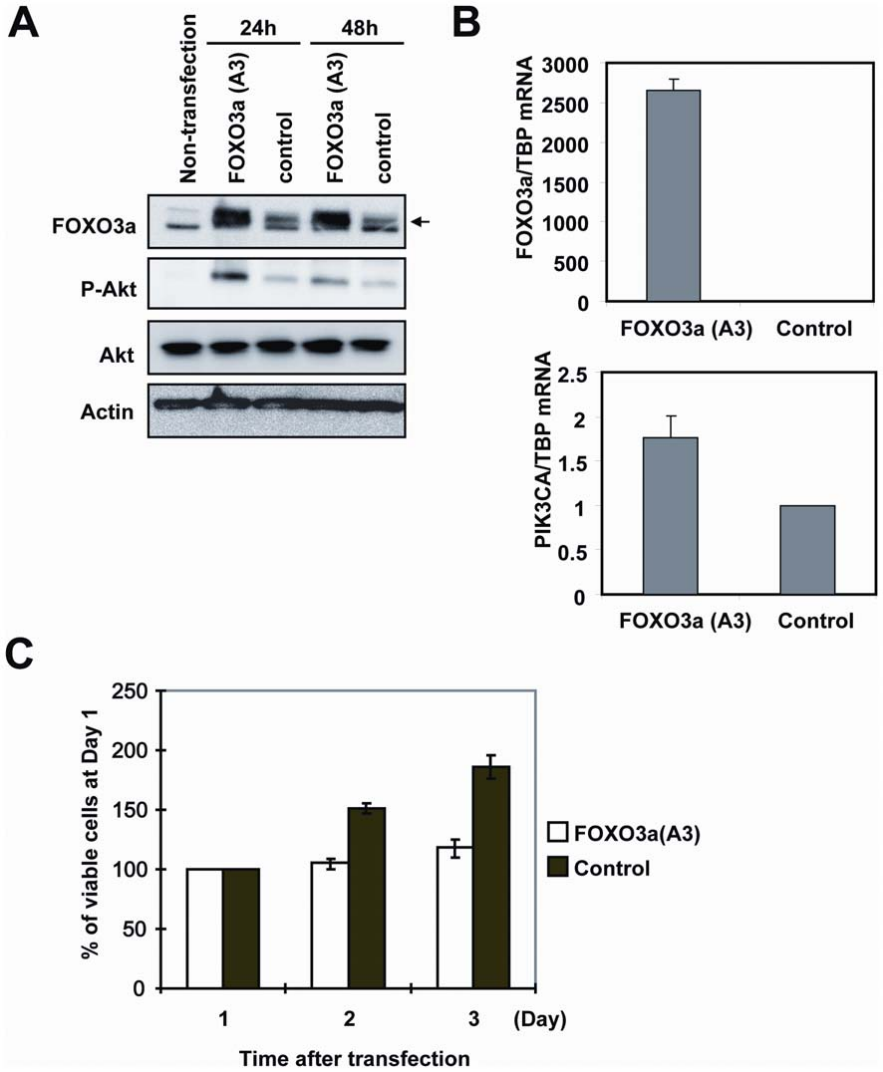
Overexpressed active FOXO3a results in cell proliferation arrest and Akt phosphorylation in drug sensitive breast cancer cell line MCF-7. MCF-7 cells were transiently transfected with the constitutively active FOXO3a(A3) or control vector, and the transfected cells analysed by A) Western blot analysis for FOXO3a, P-Akt (Ser473), Akt and β-actin. B) The transfected cells were also examined by qRT-PCR analysis for FOXO3a and PIK3CA mRNA expression. TBP mRNA was used as an internal control. C) SRB assays were performed on these transfected cells, indicating that the ectopic expression of FOXO3a(A3) decreases the cell proliferation rate.

To examine the role of FOXO3a in drug response, we examined the effects of overexpression of FOXO3a in the sensitive cell line MCF-7. The drug sensitive MCF-7 cells were transiently transfected with an expression vector encoding a constitutively active FOXO3a(A3), in which all three Akt-phosphorylation sites were mutated to alanine. The results revealed that ectopic FOXO3a(A3) expression not only enhances Akt phosphorylation without altering total Akt expression levels (Fig. 8A), but also attenuated proliferation (Fig.8C). This is consistent with a previous report using chronic myeloid leukaemia (CML) cell lines showing that FOXO3a overexpression can result in an increase in PI3K-Akt activity [23]. The gene PIK3CA, which encodes the PI3K catalytic subunit, has previously been demonstrated to be a direct downstream target of transcription factor FOXO3a in CML [23], [24]. Our experiments showed that ectopic expression of FOXO3a(A3) could enhance the expression of PIK3CA transcriptionally (Fig. 8B). Furthermore, ectopic expression of FOXO3a(A3) could also block cell proliferation in the MCF-7, suggesting that doxorubicin inhibition of cell proliferation is at least in part through FOXO3a in the cytotoxic chemotherapeutic resistant breast cancer cells.

**Figure 9:**
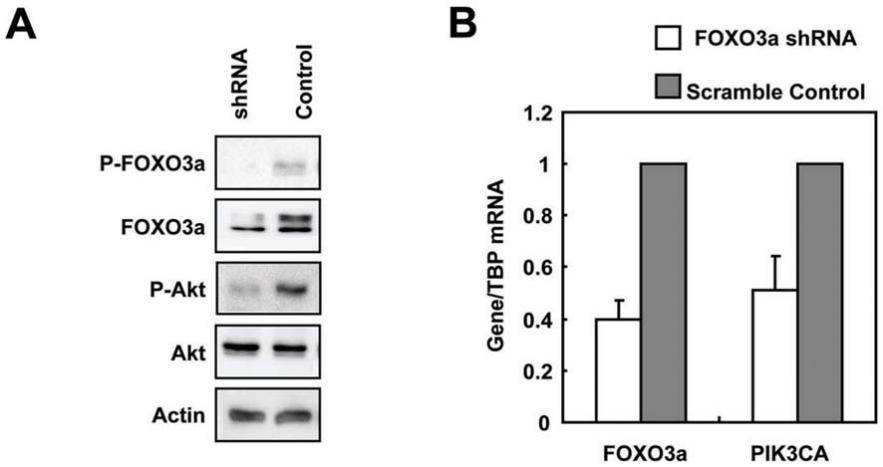
Knockdown of FOXO3a expression decreases Akt phosphorylation and PIK3CA mRNA expression. BT474 cells were transiently transfected with FOXO3a or control shRNA, and 24 h after transfection cells were analysed by A) Western blot using specific antibodies P-FOXO3a (Thr32), FOXO3a, P-Akt (Ser473), Akt and Actin as indicated and by B) qRT-PCR.

To confirm the observation that FOXO3a regulates Akt activity, specific shRNAs were used to knockdown the endogenous FOXO3a expression in the doxorubicin sensitive BT474 cell line which exhibits high PI3K-Akt activity. The knockdown effect of FOXO3a shRNAs in BT474 was confirmed by Western blot analysis (Fig. 9A) and at mRNA levels using Real-time quantitative (RTq)-PCR (Fig. 9B). FOXO3a knockdown induced a corresponding reduction in the levels of PIK3CA transcripts (Fig. 9B) and P-Akt (Ser-473) (Fig. 9A).

### FOXO3a activation promotes Akt phosphorylation but not proliferation arrest in drug resistant breast cancer cells

**Figure 10:**
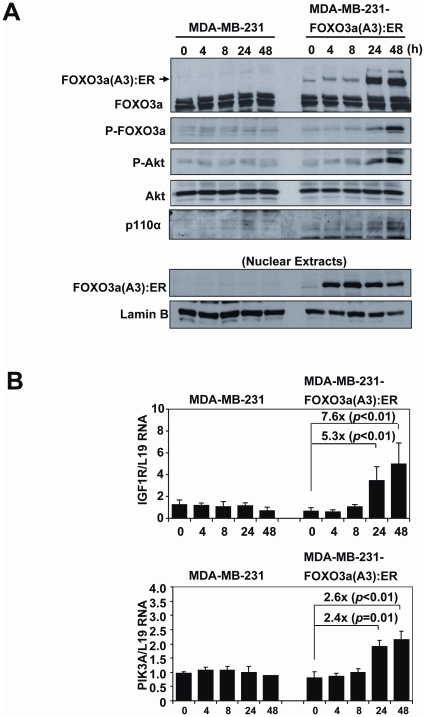
FOXO3a induces Akt phosphorylation, PIK3CA and IGFR1 gene expression in the drug resistant MDA-MB-231 breast carcinoma cells. MDA-MB-231-FOXO3a(A3):ER and MDA-MB-231 cells were treated with 200 nM 4-OHT for the indicated times. A) Total, cytoplasmic and nuclear extracts were prepared at the times indicated, separated on polyacrylamide gels, and subjected to immunoblotting with specific antibodies. The expression levels of FOXO3a, P-FOXO3a (Thr-32), p110α (PIK3CA), P-Akt (Ser473), Akt, and lamin B1 were analyzed by Western blotting. B) Total RNA was extracted from these cells and analyzed for PIK3CA, and IGFR1, mRNA expression using qRT-PCR as described in the text and normalized to the level of L19 RNA. All data shown represent the averages of data from three experiments, and the error bars show the standard deviations.

**Figure 11:**
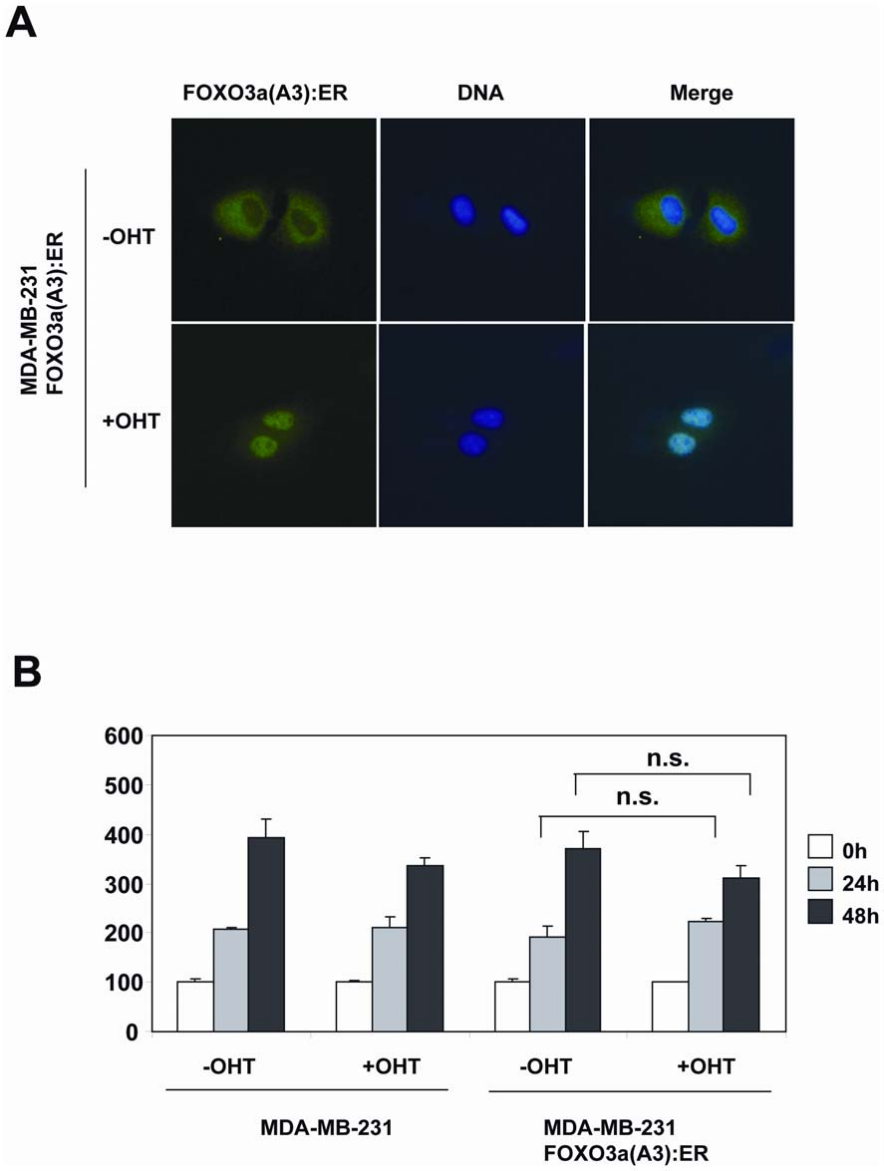
FOXO3a induction does not result in proliferative arrest in the drug resistant MDA-MB-231 breast carcinoma cells. A) MDA-MB-231-FOXO3a(A3):ER cells were cultured on sterile coverslips and treated for 16 h with or without 200 nM 4-OHT as in Fig. 10, before being fixed in 4% formaldehyde. FOXO3a(A3):ER was visualized with a rabbit polyclonal antibody against ERα followed by the addition of ALEX488 (green) labeled anti-rabbit antisera. DAPI (blue) were also applied to visualize the nuclei. B) SRB assays were performed on these cytotoxic resistant MDA-MB-231 cells, indicating that the induction of FOXO3a(A3) has little effects on the cell proliferation rate of the drug resistant breast carcinoma cells.

To study the effects of FOXO3a activation in drug resistant breast cancer cells, we used a MDA-MB-231 cell line harbouring an expression vector that encodes the hormone binding domain of estrogen receptor α (ERα) fused to the constitutively active FOXO3a(A3). In these MDA-MB-231-FOXO3a(A3):ER cells, FOXO3a activity can be conditionally induced upon the addition of Tamoxifen (4-OHT). Western blot analysis showed that treatment of MDA-MB-231-ER:FOXO3a(A3) cells with 4-OHT induced the relocation of FOXO3a from the cytoplasm to the nucleus fractions (Fig. 10A). The result also showed that the relocation of FOXO3a(A3):ER to the nucleus upon 4-OHT treatment enhanced Akt phosphorylation without significantly altering total Akt expression levels. This increase in Akt phosphorylation, and thus PI3K-Akt activity, was specifically mediated by FOXO3a induction, as no such response was observed in the control MDA-MB-231 cells expressing ER alone. Consistent with this, RT-qPCR analysis also showed that treatment with 4-OHT also induced the expression of the FOXO3a target genes, PIK3CA and IGFR1 (Fig. 10B). However, only p110α the gene product of PIK3CA, could be detected at protein levels in these cells upon FOXO3a induction (Fig. 10A). The nuclear relocation of FOXO3a(A3):ER in response to 4-OHT induction in the MDA-MB-231-FOXO3a(A3):ER cells was confirmed by immunoflorescence staining of the estrogen receptor (ER) (Fig. 11A). Notably, the induction of FOXO3a activity in the drug resistant MDA-MB-231 cells did not cause significant changes in the rate of cell proliferation (Fig. 11B), indicating that the anti-proliferative function of FOXO3a is deregulated in the cytotoxic drug resistant cells. This might help to explain why the breast cancers of poor prognosis can tolerate nuclear FOXO3a.

### FOXO3a function is deregulated in the drug resistant breast cancer cells

**Figure 12:**
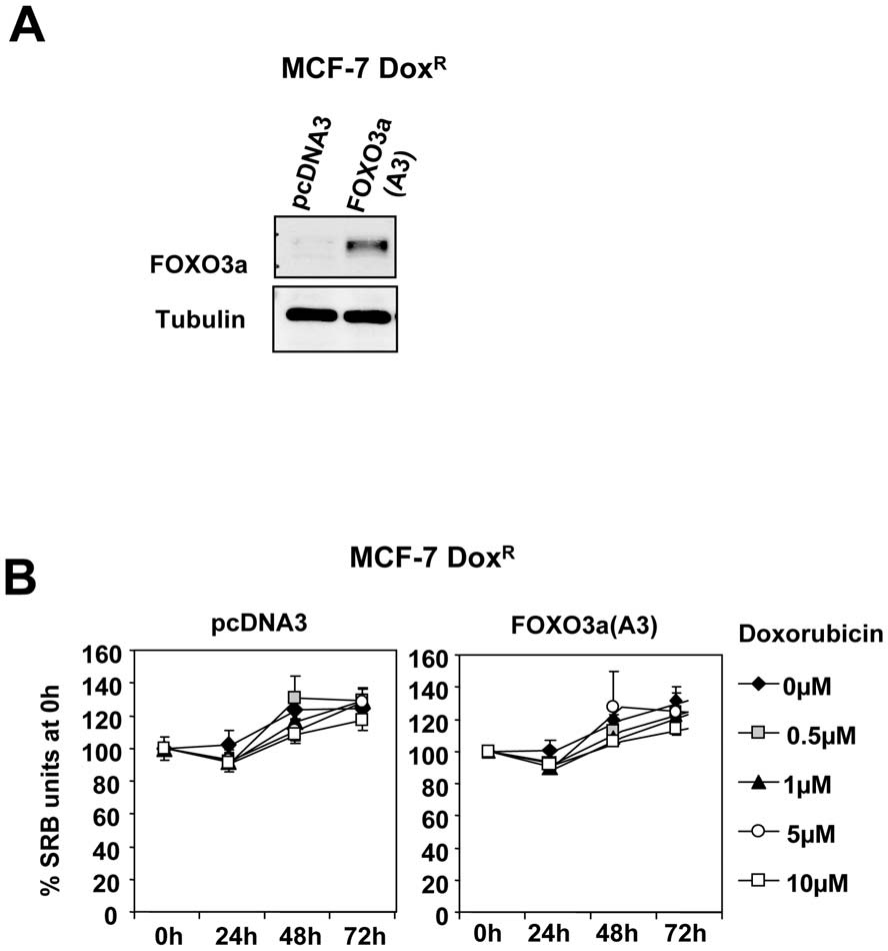
Overexpressed active FOXO3a has little effect on cell proliferation and drug sensitivity of the drug resistant breast cancer cell line MCF-7-Dox^R^. MCF-7-Dox^R^ cells were transiently transfected with the constitutively active FOXO3a(A3) or control vector pcDNA3, A) The transfected cells were analysed by Western blot analysis for FOXO3a and β-actin expression. B) The cells were treated with 0 to 10 µM of doxorubicin for 0, 24, 48 and 72 h and SRB assays were performed on these transfected MCF-7-Dox^R^ cells after 48 h. The results indicated that the induction of FOXO3a(A3) has little effect on the cell proliferation rate of the drug resistant breast carcinoma cells and in response to doxorubicin treatment.

**Figure 13:**
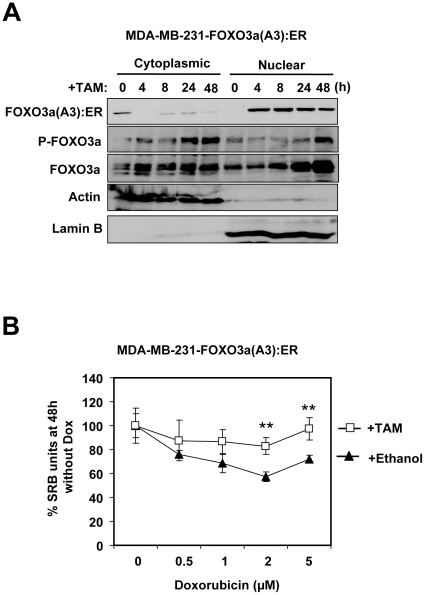
Induction of FOXO3a can confer resistance to the MDA-MB-231 cells. MDA-MB-231-FOXO3a(A3):ER cells were cultured with 200 nM 4-OHT and collected at 0, 4, 8, 24 and 48 h. A) Total cytoplasmic and nuclear extracts were prepared at the times indicated, separated on polyacrylamide gels, and subjected to immunoblotting with FOXO3a, P-FOXO3a(Thr-32), actin and lamin B1 antibodies. B) SRB assays were performed on the untreated and 4-OHT-treated MDA-MB-231 cells after 48 h of treatment with various concentrations of doxorubicin. The results indicated that the induction of FOXO3a (A3) can protect the drug resistant breast carcinoma MDA-MB-231 cells from the effects of doxorubicin.

To confirm further that the anti-proliferative function of FOXO3a is deregulated in the cytotoxic drug resistant cells, the doxorubicin resistant MCF-7Dox^R^ cells were transfected with either the empty expression vector or one that encodes for the constitutively active FOXO3a(A3) (Fig. 12A) and cultured with various doses of doxorubicin. The proliferative assays demonstrated that overexpression of the active FOXO3a(A3) did not result in any significant decreases in proliferative rate (Fig. 12B), suggesting that the anti-proliferative function of FOXO3a is deregulated in the cytotoxic drug resistant cells. This result also argues against the possibility that FOXO3a mutation is the predominant cause of drug insensitivity in the drug resistant cells. To examine further the effects of FOXO3a activation on drug resistance in breast cancer cells, the MDA-MB-231-FOXO3a(A3):ER cells were treated with either the vehicle (ethanol) or 4-OHT and subjected to doxorubicin treatment. Western blot analysis of cytosolic and nuclear protein fractions showed increased nuclear and decreased cytoplasmic FOXO3a levels after 4 h of tamoxifen treatment, implying increased FOXO3a transcriptional activity (Fig. 13A). FOXO3a activation also resulted in an increase in Akt activity, as revealed by the increased in P-FOXO3a (Thr-32) expression. Notably, at 24 and 48 h after FOXO3a(A3):ER induction, there was an accumulation of endogenous FOXO3a, which can probably help to explain for the late induction in Akt activity as well as the expression of FOXO3a targets. Proliferative assays demonstrated that high levels of doxorubicin significantly decreased the proliferation rates of the vehicle-treated MDA-MB-231-FOXO3a(A3):ER cells, indicating that these cells are sensitive to high levels of doxorubicin (Fig. 13B). Interestingly, the results also showed that activation of FOXO3a(A3):ER by 4-OHT can confer resistance to the anti-proliferative effects of high levels of doxorubicin in the MDA-MB-231-FOXO3a(A3):ER, further confirming that FOXO3a function is deregulated in the drug resistant breast cancer cells.

## Discussion

Tamoxifen and cytotoxic chemotherapy are commonly used for the treatment of breast cancer patients worldwide. In breast cancer, numerous endocrine therapeutic strategies have been developed to target the estrogen and the downstream ERα-signaling cascade. Of these endocrine therapeutic agents, tamoxifen is the most commonly used anti-estrogen [1], [3], [4], [5]. Anthracyclines and taxanes, used alone or in combination, are the most prevalent cytotoxic agents for the treatment of breast cancer and in particular metastatic diseases. The PI3K-Akt signaling pathway plays a vital role in tamoxifen and cytotoxic chemotherapeutic drug resistance [10], [11], [12], [13]. Enhanced Akt activity has been reported to promote resistance to tamoxifen and cytotoxic drugs by promoting cell proliferation and survival [25]. The transcription factor FOXO3a is one of the key effectors of the PI3K-Akt signaling pathway. FOXO3a can be phosphorylated by Akt, resulting in its inactivation and nuclear exclusion. The Akt-FOXO3a axis also mediates the cytotoxic response of chemotherapeutic drugs, including anthracyclines, (eg. doxorubicin also called Adriamycin) and taxanes (eg. paclitaxel also called Taxol) and cisplatin [21], [22], [23], [24], [26], [27], [28], [29]. Paclitaxel induces the nuclear translocation and accumulation of FOXO3a to induce apoptosis in sensitive breast cancer cell lines [22], [27]. In colon carcinoma cells, cisplatin also promotes the nuclear accumulation of FOXO3a to induce cell death [26]. Similarly, in CML cells, the cytotoxic effect of doxorubicin is also mediated by the nuclear accumulation of FOXO3a [23], [24]. However, sustained FOXO3a activation by doxorubicin doxorubicin can also promote drug resistance and survival of leukaemic cells by activating the expression of genes important for drug efflux (eg. ABCB1) and survival (eg. PIK3CA) [23], [24], [30].

In a study of 113 breast cancer cases, Hu *et al* [16] have previously reported that FOXO3a is localized mainly in the cytoplasm of breast cancer cells and that predominant cytoplasmic FOXO3a localization is inversely associated with patient survival. Contrary to these findings, our immunohistochemistry results showed that nuclear FOXO3a expression in invasive ductal carcinoma is associated with lymph node metastasis (p = 0.052) and shortened overall survival time (p = 0.014). These data also indicate that consistent nuclear FOXO3a expression in patient samples is associated with shorter survival time after treatment, as most if not all of these patients were treated and monitored after diagnosis. Nevertheless, our findings are in keeping with another report showing that predominant nuclear-targeted FOXO3a is significantly correlated with lymph node metastasis in invasive ductal breast carcinoma [17]. Furthermore, using the same scoring system as that of Hu et *al* [16], we found that cases with higher nuclear to cytoplasmic FOXO3a expression ratio are also positively associated with P-Akt expression (p = 0.039, Chi-square test). Consistent with our findings, predominant nuclear expression of FOXO3a have also been shown to correlate with enhanced PI3K/Akt activity and acquisition of chemoresistance in chronic myeloid leukaemia (CML) cell lines [23], [24].

In the present study, FOXO3a subcellular localization was investigated in a panel of endocrine and cytotoxic chemotherapy resistant and sensitive breast cancer cell lines to establish the significance of our findings *in vivo* and to test the hypothesis that active FOXO3a is associated with resistance to chemotherapy. Similar to that observed in doxorubicin-resistant CML cell lines [23], [24], our tissue culture models showed enhanced basal phosphorylated Akt in tamoxifen-resistant cell lines compared with the parental sensitive cell line MCF-7. Interestingly, high levels of nuclear FOXO3a were only detected in doxorubicin (eg. MCF-7Dox^R^ and MDA-MB-231) but not tamoxifen (eg. LCC2, LCC9 and BT474) resistant breast cancer cell lines. In terms of therapeutic options for breast cancers, although the overall prognosis associated with tamoxifen resistant breast cancers is usually poor, these breast cancers may still respond to other endocrine (eg. Fulvestrant and aromatase inhibitors, including anastrozole, letrozole and exemestane) as well as cytotoxic chemotherapy treatments. In fact, approximately 30% of ERα positive breast patients do not respond to tamoxifen treatment, which is defined as *de novo* resistance. Moreover, the majority of tumours that initially respond to tamoxifen treatment will acquire resistance over time even with maintained ERα expression [31], [32]. Yet, most of these *de novo* or acquired endocrine resistant breast cancers are at least initially sensitive to the cytotoxic chemotherapy. Consistent with this notion, all the tamoxifen-resistant cell models tested in the present study, including LCC2, LCC9, BT474, are sensitive to doxorubicin treatment, whereas the doxorubicin resistant MCF-7Dox^R^ and MDA-MB-231, are not sensitive to tamoxifen. Notably, the MDA-MB-231 cells are also resistant to the taxane paclitaxel treatment [22], [27]. As a result, these tamoxifen-resistant breast cancer cells may model breast cancers that have failed endocrine therapy but remain sensitive to cytotoxic drug-based treatments, while the resistant cells may represent breast cancers that have progressed and become resistant to anthracyclins and taxanes. These drug-resistant, advanced breast cancers are of worse prognosis, as their treatment options are very limited. The cytotoxic chemotherapeutic resistant cell lines and the invasive ductal breast carcinoma have numerous significant overlapping clinical and pathological features. In this study, we show that they at least share a common molecular phenotype of sustained nuclear FOXO3a expression. Taken together our data from the cell culture models help to support and explain our immunocytochemistry findings that in advanced breast cancers FOXO3a is predominantly nuclear localized despite the presence of phophorylated Akt. This also provides *in vitro* evidence suggesting sustained nuclear FOXO3a expression predicts poor survival and chemotherapy resistance.

Our *in vitro* cell culture experiments confirmed that overexpression of active FOXO3a(A3) in both MCF-7 and MDA-MB-231 can indeed enhance Akt activity, supporting the feedback mechanism via the PI3K/Akt pathway. However, overexpression of the active FOXO3a(A3) can efficiently inhibit cell proliferation in the chemosensitive breast cancer MCF-7 but has little effect in the resistant MDA-MB-231 cells. Previously, FOXO3a has also been shown to induce the expression of the cell cycle inhibitor p27^Kip1^ and the pro-apoptotic Bcl-2 protein Bim to induce proliferation arrest and apoptosis, respectively, in MCF-7 breast cancer cells. In line with this, doxorubicin treatment of the sensitive MCF-7 cells also resulted in re-localization of FOXO3a to the nucleus in our studies. Thus the enhanced P-Akt expression resulting from FOXO3a induction in MCF-7 is insufficient to counteract the action of activated FOXO3a, resulting in an overall decrease in cell viability. However, in the chemoresistant cell line MDA-MB-231, the chemoresistant cells may have already adopted as yet unknown mechanisms to avoid the anti-proliferative effects of activated FOXO3a, remaining unresponsive to cell cycle arrest and apoptosis under drug treatment.

The sustained nuclear localization of FOXO3a may also contribute to the increased PI3K-Akt activity observed in the chemoresistant breast cancer cells. Treatment of MDA-MB-231-FOXO3a:ER cells but not the MDA-MB-231 with 4-OHT was sufficient to induce P-Akt expression, suggesting that the increase in FOXO3a activity is causative for the induction of PI3K-Akt activity. Moreover, induction of FOXO3a activity also resulted in the induction of expression of PIK3CA and IGF-R1, two components of the PI3K-Akt signaling cascade. The increase in expression levels of PIK3CA and IGF-R1 as well as other components of PI3K-Akt signaling pathway by FOXO3a may be sufficient for the induction Akt phosphorylation and activity if their expression levels are limiting in these breast cancer cells.

FOXO3a activity is likely to be deregulated in both the invasive ductal breast carcinoma associated with poor survival, as well as the anthracyclin-resistant breast cancer cell lines as in both cell-types nuclear-localized FOXO3a failed to elicit its anti-proliferative effects. Consistently, induction of FOXO3a activity and nuclear localization using 4-OHT in the drug-resistant MDA-MB-231-FOXO3a(A3):ER cells did not have an appreciable effects on the cell proliferation. Furthermore, expression of an active form of FOXO3a can enhance the resistance to doxorubicin in the MDA-MB-231, particular at higher doses. The molecular basis for this is unclear but can be due to post-translational modifications and/or differential recruitment of co-factors. Nevertheless, sustained nuclear localization of FOXO3a may be a crucial molecular marker for chemotherapy resistance and poor prognosis in breast cancer. Consistent with the idea that overexpression of FOXO3a can contribute to tumour progression, a recent study showed that FOXO3a promotes tumour cell invasion and metastasis through the induction of matrix metalloproteinases. As such, depletion of FOXO3a from cancer cells results in decreased tumour size as a result of attenuated invasive migration [33]. Together these data suggest that lymph node metastasis and poor survival in invasive ductal breast carcinoma are linked to an uncoupled of the Akt-FOXO3a signaling axis, as in these breast cancers active Akt fails to inactivate and re-localize FOXO3a to the cytoplasm and that nuclear-targeted FOXO3a does not induce cell death or cell cycle arrest.

In summary, sustained nuclear FOXO3a expression in breast cancer may culminate in cancer progression and the development of an aggressive phenotype similar to that observed in cytotoxic chemotherapy resistant breast cancer cell models and, can significantly attenuate their response to tamoxifen and cytotoxic chemotherapy. Studying the expression profiles of the components of the Akt-FOXO3a axis, in breast cancer patients might help predict and monitor their response to chemotherapy. Further investigations will be required to determine the mechanism by which FOXO3a nuclear localization and Akt activity are uncoupled in resistant cancer cells. A better understanding of the mechanism by which Akt and FOXO3a are regulated, as well as their roles in cancer progression, drug sensitivity and resistance, may turn these proteins into crucial therapeutic targets and prognostic markers for breast cancer and other malignancies.
